# Examination of phacoemulsification tips after different numbers of cataract surgeries

**DOI:** 10.1038/s41598-024-67891-0

**Published:** 2024-07-24

**Authors:** Agnes Revak, Gabor Nemeth, Judit Korizs, Gergo Gyulai, Agnes Abraham, Eva Kiss, Zoltan Sohajda

**Affiliations:** 1 Department of Ophthalmology, Clinical Centre Kenézy Gyula Campus, University of Debrecen, Bartók Béla Út 2-26, Debrecen, 4031 Hungary; 2 Faculty of Health Sciences, University of Miskolc, Szentpéteri Kapu 72-76, Miskolc, 3526 Hungary; 3 Laboratory of Interfaces and Nanostructures, Eötvös Loránd University, Pázmány Péter Sétány 1/A, Budapest, 1117 Hungary

**Keywords:** Phacoemulsification tips, Microscopic damage, Multiple-use, Single-use, Medical research, Lens diseases, Eye diseases

## Abstract

To compare unused phacoemulsification tips and those used different times with different techniques of cataract surgery (divide and conquer and chop), in vivo phacoemulsifications were performed with tips of different numbers of operation. These were compared with the same number of sterilized-only and unused tips with the help of an atomic force microscope. Comparison of roughness values (Sa, Sq), geometric and measurable flange length and surface was also performed (profile length %, area %). The differences between the parameters that can be measured during surgery (average ultrasound percentage, US ave %, Average Phaco Time, APT) were also analyzed. We found significant correlations between age and lens hardness (p = 0.0045), area % and APT (p = 0.03), between area % and US ave% (p = 0.03) and also between the two surgical techniques in terms of area% (p = 0.04) and US ave % (p < 0.01). Roughness increased with the number of uses. An increase in profile length% can be observed up to the twentieth operation. This can result from scratches and microscopic damages and also from abrasion and possible material additions on the surface of the needles. The divide and conquer technique causes less microscopic damage to the surface, and smaller average US energy is required during surgery.

## Introduction

Nowadays, cataract surgery is the most commonly performed ophthalmic intervention, and its modern form is phacoemulsification. These surgeries require special equipment, for which there are several alternatives based on the manufacturer’s recommendations. Operating physicians currently use single or multiple-use phacoemulsification tips during operations. In countries where it is legally allowed, mostly due to financial reasons, the single-use tips are used more than once (which is off-label), and the number of uses of the multiple-use tips in practice is higher than recommended by the manufacturer^1–3^. In this study, we are looking for an answer to the question of how much the number of surgeries affects the abrasion of the tips, and whether it is possible to determine a limit for the use of the tips which would not result in a significant change in the morphology of the tips, and therefore would have no influence on the outcome and complications of the surgery. Furthermore, we also performed a comparison of the two currently most common surgical techniques (divide and conquer, chop) in terms of their effects on the phacoemulsification tips. In the course of this study, we examined and compared unused, sterilized-only, single-use and multiple-use phacoemulsification tips that underwent different number of surgeries based on atomic force microscopy and reflection light microscopy.

## Methods

### Patients

In our study, patients between the ages of 22 and 94 diagnosed with grade I-IV cataracts were included. The population was divided into two groups, one group was operated with the divide and conquer technique and the other with the chop technique. The operations were preceded by a comprehensive ophthalmological examination (visual acuity examination, intraocular pressure measurement, slit-lamp examination with narrow and dilated pupils, fundus examination) and intraocular lens planning. Then the operations were performed under topical anaesthesia.

The examinations and operations were carried out within the framework approved by the local ethics committee (Regional and Institutional Research Ethical Committee of the University of Debrecen) (BORS-12/2020, DEKGYEK, 10/2020).

All procedures performed in this study involving human participants were in accordance with the ethical standards of the University of Debrecen, and with the 1964 Helsinki Declaration and its later amendments or comparable ethical standards.

### Phacoemulsification tips

During our study, cataract surgeries were performed with Bausch and Lomb DP8230A (bent multiple-use) and Bausch and Lomb DP8230S (straight, single-use) phacoemulsification tips. For both tips, the measurements were made in the factory condition and after five, ten, twenty, thirty, fifty and ninety sterilization cycles. After performing the same number of surgeries, we also performed the measurements.

### Surgical techniques

The surgeries were performed by two experienced physicians, one of them used divide and conquer, the other used the horizontal chop technique, so it was possible to compare the effects of these techniques on the phacoemulsification tips. During the operations, the age of the patients, grade of the cataract (grade I-IV), duration of the operation (case time), average phaco time (APT), effective phaco time (EPT), average phaco energy (US AVE%), the possible contact between the phaco tip and the auxiliary device during surgery were analyzed, as well as the possible surgical complications.

### Sterilization

The phacoemulsification tips were cleaned each time with distilled water and compressed air at a pressure of 2 bar. Sterilization was then carried out using an autoclave, unpacked, for 3.5 min at 134 °C.

### Morphological examination

In this study, we used atomic force microscopy (AFM) and reflection light microscopy to examine the morphology of the phacoemulsification tips. With the help of light microscopy, we can obtain qualitative information about the abrasion of the edge and mantle of the tips. Atomic force microscopy can also be used to quantitatively monitor the morphological changes occurring at the tip and the mantle of the needles through the determination of the surface roughness. The measurements were performed on two parts of the needles: at the very end of the tip of the needles, which first comes into contact with the tissues (edge) and on the part of the outer mantle of the needles close to the tip (~ 50–100 μm distance) (mantle). At these locations, topographic images of 20 × 20 μm^2^ areas were taken using AFM. In addition, light microscopic images were taken from the edge around and from an area of approximately 1 mm on the mantle.

For the AFM measurements, we used a Park XE-100 device, in tapping mode, with an NSC15 probe, at a resolution of 512 × 512 pixels. After evaluating the recorded images, we performed a roughness analysis. In the course of this, we determined the average surface roughness (S_a_), the root mean square surface roughness (S_q_) and the ten-point height (S_z_):

$${S}_{a}=\frac{1}{N}\sum_{j=1}^{N}\left|{z}_{j}-\overline{z }\right|$$

$${S}_{q}=\sqrt{\frac{1}{N}\sum_{j=1}^{N}{\left({z}_{j}-\overline{z }\right)}^{2}}$$

where N is the number of measurement points,$\overline{z }$ is the average height value and z_j_ is the height value at a given point.

The S_a_ value is a simple overview numerical value of the average roughness, while the S_q_ value is more sensitive to larger level differences occurring on the sample, and therefore more sensitively indicates the presence of a small number of large objects. The S_z_ value indicates the difference between the average of the 10 highest and 10 lowest points of the sample, it can be particularly sensitive if there are only localized but large level differences on the sample. The roughness values were determined according to the algorithm below. The representative AFM images were divided into 25 equal (4 × 4 μm^2^) fields. We performed the roughness analysis on the image details separately. We took the average of the obtained 25 roughness data and determined the confidence interval. Based on the size of the confidence interval, we obtained additional information about the topographic homogeneity of the samples on the 4 μm size scale.

Edge abrasion was also examined by analyzing reflection light microscope images. To do this, we determined the length of the outer edge of the needle's edge, and then compared it to the length of the ideal elliptical profile. We also examined the change in the area of the edge of the needle. A circle was fitted to the outer edge of the needle tip and a mask was produced using the fitted profile. The length of the arc and the area of the mask were determined from the image. The length of the resulting arc (l_id_) was compared to the ideally smooth edge line length. We also added a circular arc to the inner edge of the needle tip and created a mask in this way. We also determined the area of this mask, and, as the difference between the two masked areas, we obtained the area of the article of the ideal needle (A_id_). After that, the image was converted to binary after setting appropriate limits. We determined the length of the profile (l) and the area of the needle section (A) on the outer curve.

The obtained values are compared to the ideal curve values (l/l_id_·100% and A/A_id_·100%).

### Statistical analysis

Statistical analysis was conducted using Microsoft Excel software (version 2016, Microsoft Corp.). Descriptive statistics were computed, encompassing measures such as means, standard deviations (SDs), and measurement ranges. Additionally, the normality of the data was examined through the application of the Kolmogorov–Smirnov test. Wilcoxon test was used to compare the data between groups and the assessment of correlations between variables was performed using the Spearman rank test.

### Ethics approval

All procedures performed in this study involving human participants were in accordance with the ethical standards of the University of Debrecen, and with the 1964 Helsinki Declaration and its later amendments or comparable ethical standards.

### Consent to participate

Authors confirms that informed consent was obtained from all participants and/or their legal guardians.

## Results

During our study, 2 surgeons performed unilateral cataract surgery on 820 eyes. Average age of patients was 69.67, and the mean of cataract grade was 2.65. There was no significant difference in cataract grade between the two groups of patients operated with different surgical techniques (p = 0.52). The average grade of cataract was 2.58 in the case of the divide and conquer technique, and 2.70 of patients who were operated with the chop technique.

**Figure 1:**
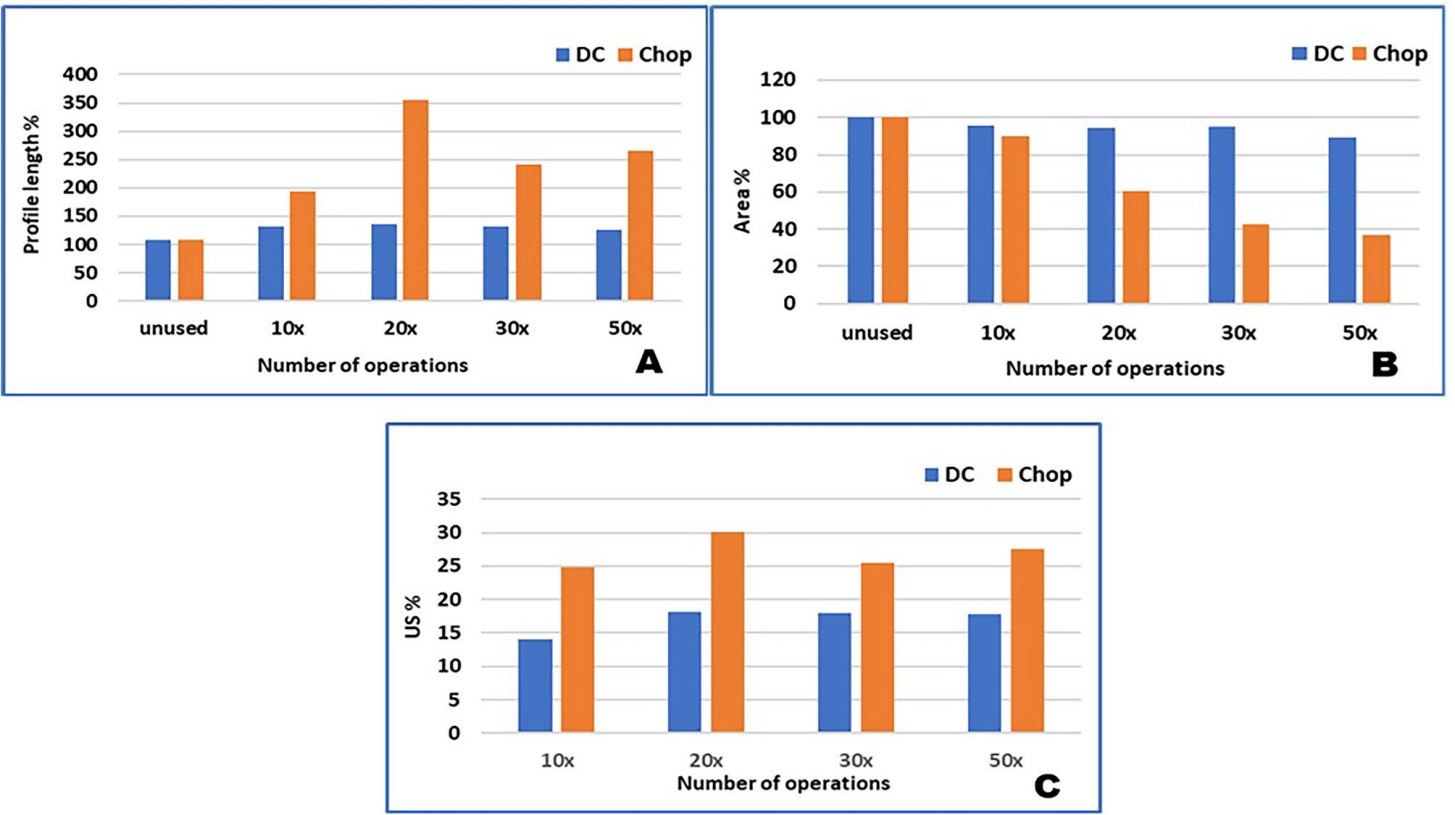
Differences between divide and conquer and chop technique after different numbers of operation. (**A**): Profile length percentage differences. (**B**): Area percentage differences (**C**): Average US percentage differences. *DC* Divide and conquer.

**Figure 2:**
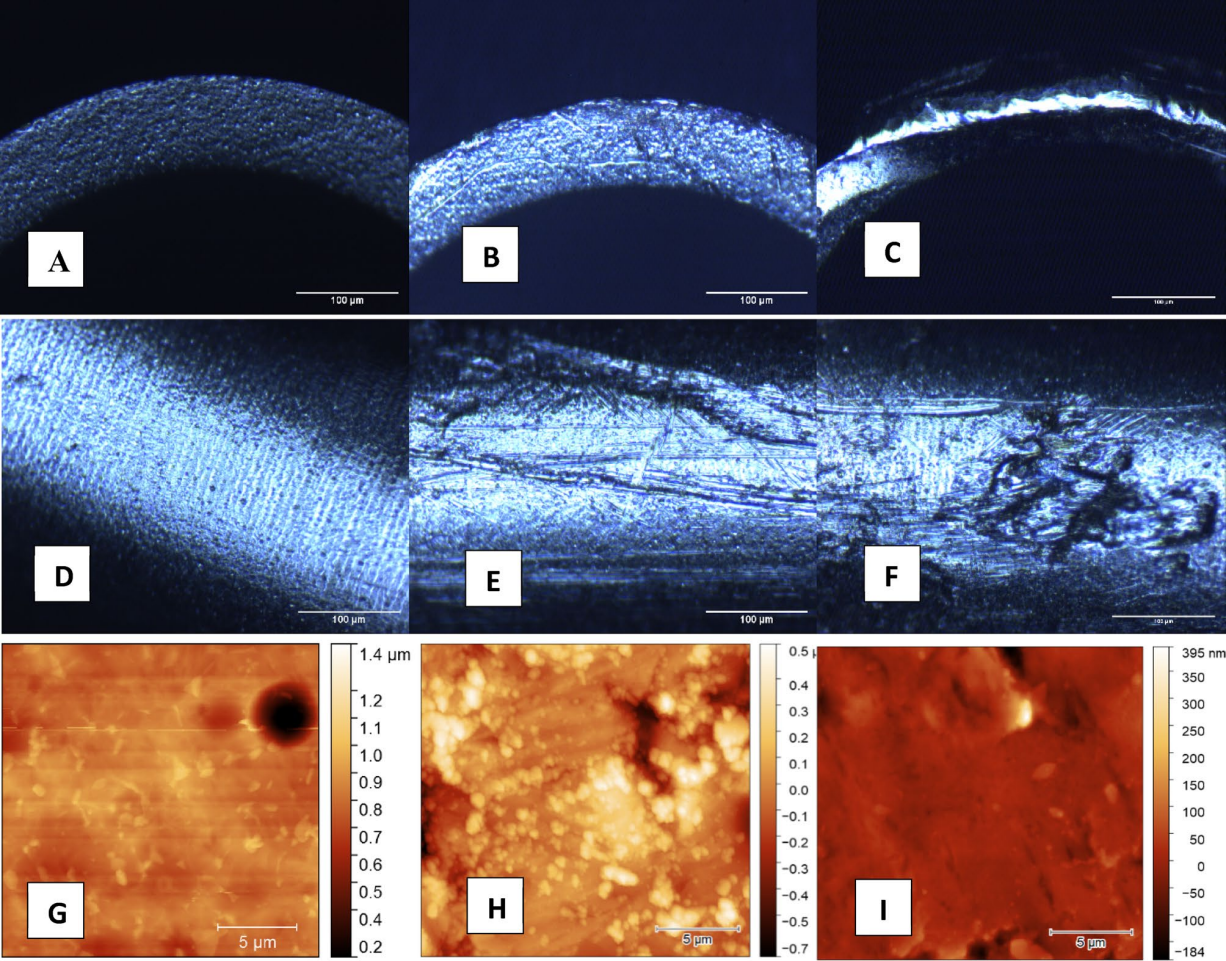
(**A**,**B**,**C**): Microscopic changes on phaco tip edges after fifty operations with divide and conquer (**B**) and chop technique (**C**). (**D**,**E**,**F**): Microscopic changes on phaco tip mantles after fifty operations with divide and conquer (**E**) and chop technique (**F**). (**G**,**H**,**I**): Atomic-force microscopic changes on phaco tips after fifty operations with divide and conquer (**H**) and chop technique (**I**). A,D,G: Unused tips.

Based on the analysis of our data, significant correlations were found between age and cataract grade (r = 0.87, p = 0.0045), between area percentage and absolute phaco time (APT) (r = − 0.9, p = 0.03), and between the area percentage and US EPT (r = − 0.9, p = 0.03). According to the negative correlation between the area % and APT and EPT data, the longer the phaco energy is used, the greater the reduction of the area %, thus the abrasion of the tip will also be greater. However, in the case of profile length %, we found a significant difference in only one roughness index (r = 0.9, p = 0.03). We found a significant difference between the two surgical techniques in terms of the percentage of the area (p = 0.04) and the average US energy (US ave %) used during the operation (p < 0.01) (Figs. 1, 2).

With the exception of one roughness index (Sz_conf: p = 0.03), the figures obtained in the case of operations performed with the divide and conquer technique are higher compared to the chop technique. On average, higher ultrasound energy was required during operations performed using the chop technique. The difference in area % indicates that the surface abrasion of the tips is less with the divide and conquer technique.

Unused single-use and multiple-use tips differed in several edge and mantle parameters (Edge: Sa p = 0.04, Sq p = 0.04, Sz p = 0.02, Mantle: Sa p = 0.01, Sq p = 0, 01).

**Figure 3:**
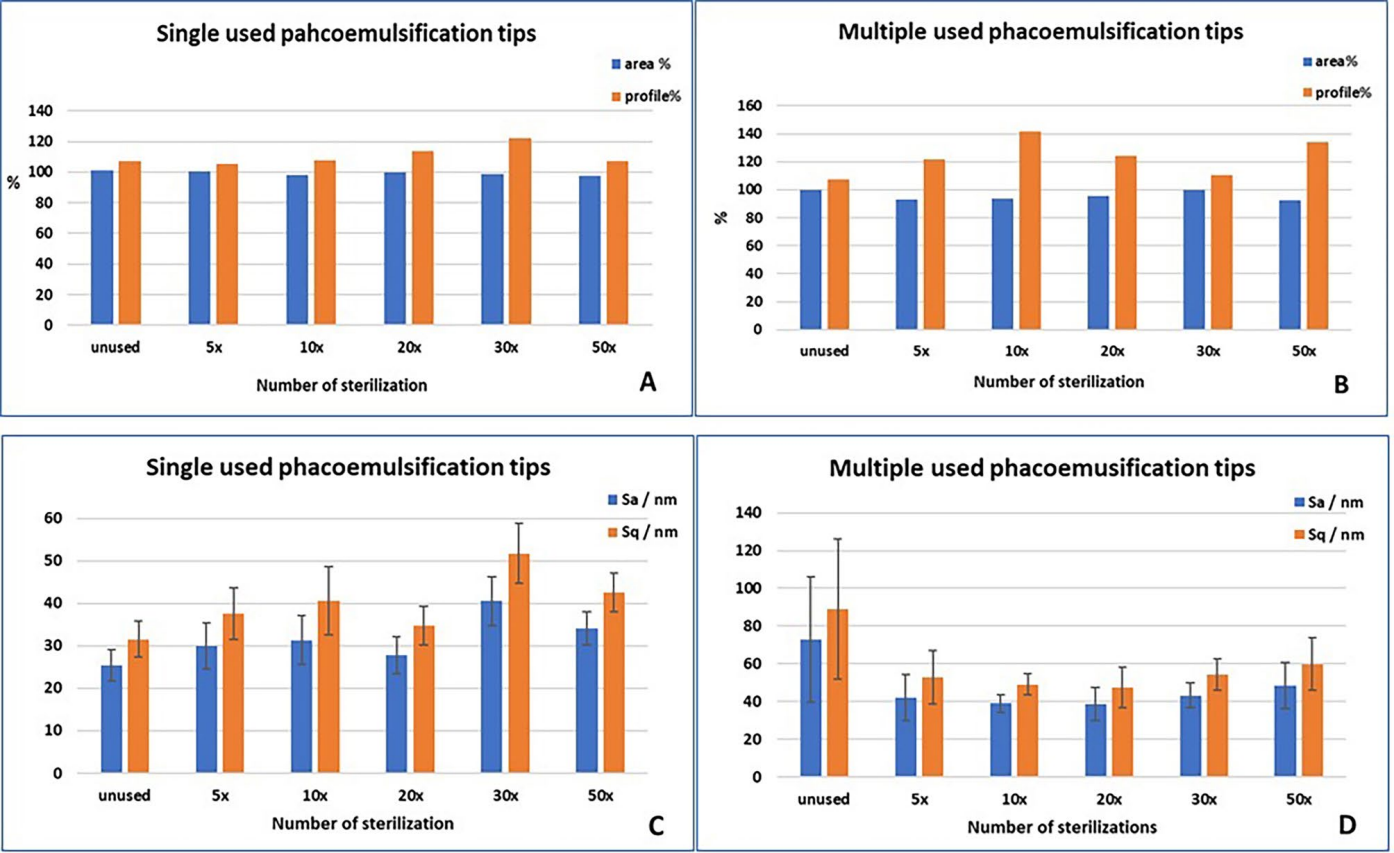
Effects of sterilization. A, B: Area and profile length percentage changes after different numbers of sterilization on single-use (**A**) and multiple-use (**B**) tips. C, D: Surface roughness changes after different numbers of sterilization on single-use (**C**) and multiple-use (**D**) tips. S_a_/nm = average surface roughness; S_q_/nm = surface mean square roughness.

**Figure 4:**
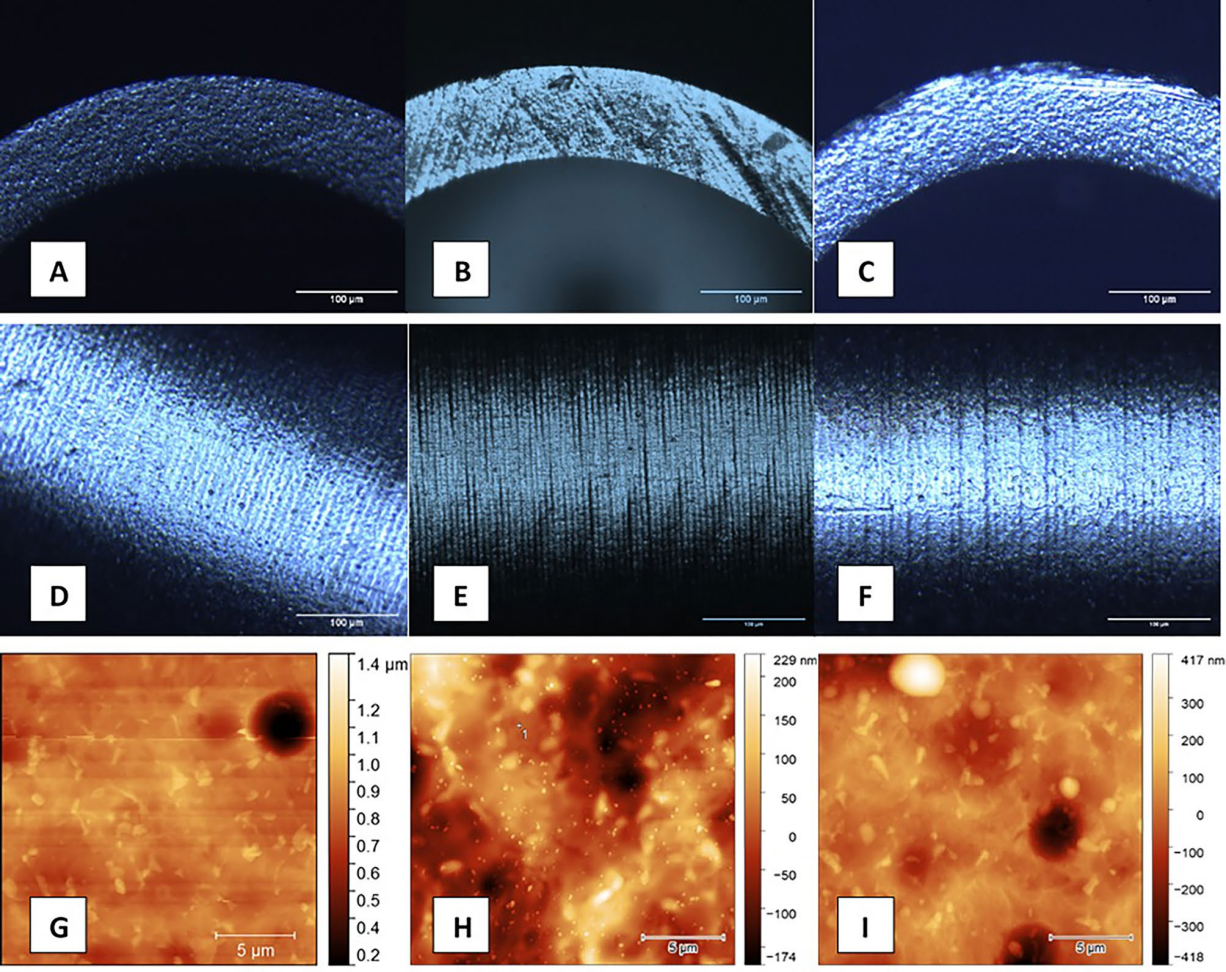
(**A**,**B**,**C**): Microscopic changes after fifty sterilization periods on the edge of a single-use (**B**) and a multiple-use tip (**C**). (**D**,**E**,**F**): Microscopic changes after fifty sterilization periods on the mantle of a single-use (**E**) and a multiple-use tip (**F**). G,H,I: Atomic-force microscopic changes after fifty sterilization periods on the edge of a single-use (**H**) and a multiple-use tip (I). A,D,G: Non-sterilized, unused tip.

Differences were also found between the sterilized-only but not used single-use and multiple-use tips in several data of the edge and mantle (Edge Sa p = 0.03, Sq p = 0.05 Sz p = 0.04, Sz and Mantle Sa p < 0.01, Sq p = 0.01, Sq p = 0.01). In the parameters of the multiple-use tips, the edge while in the values of the single-use tips, mantle indicators were higher, but sterilization had no significant effect on the parameters of the edge and the mantle (Figs. 3, 4).

**Figure 5:**
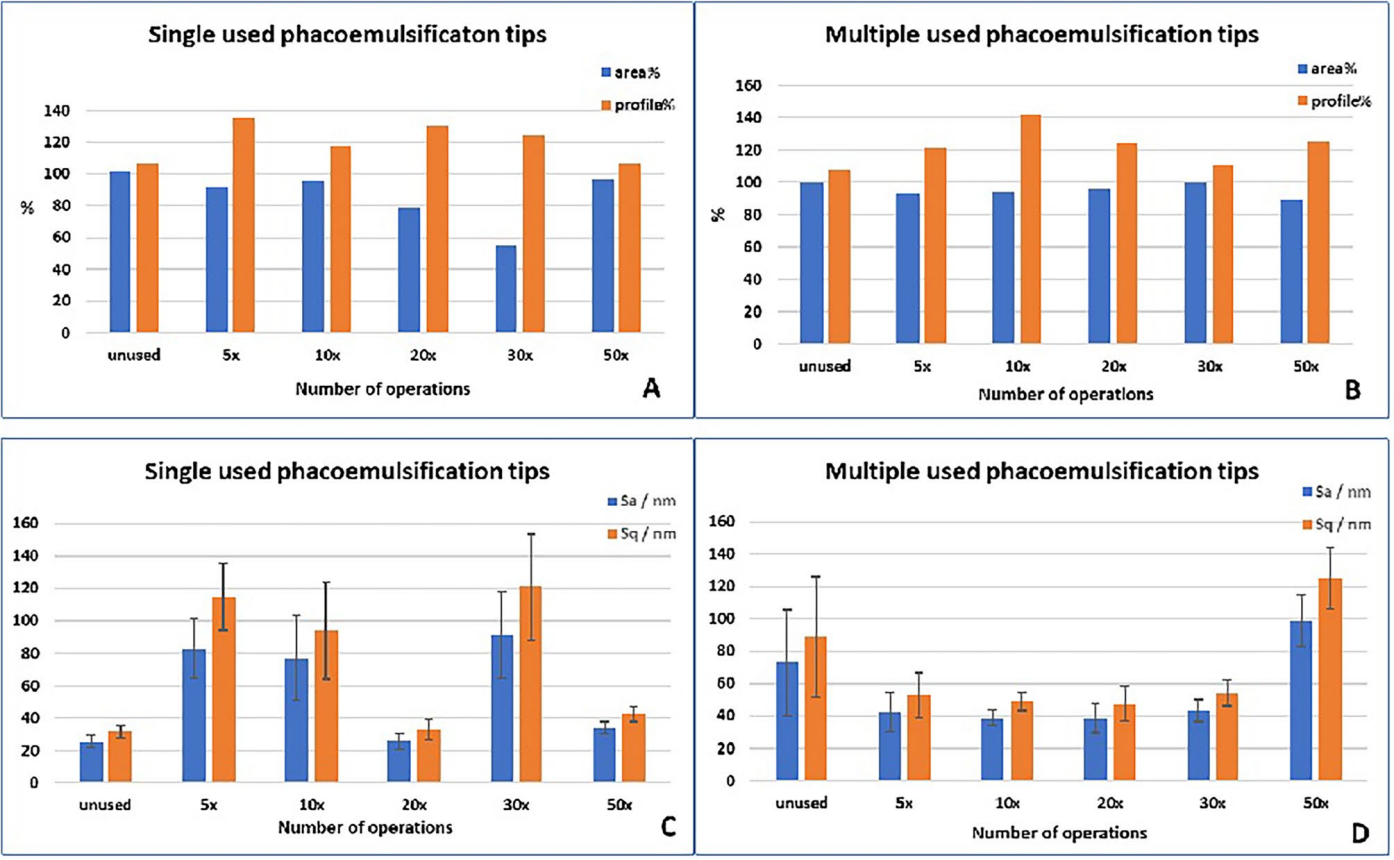
Effects of operations on the surface of single- and multiple-use phacoemulsification tips. (**A**,**B**): Area and profile length percentage changes after different numbers of operation on single-use (**A**) and multiple-use (**B**) tips. C,D: Surface roughness changes after different numbers of operation on single-use (**C**) and multiple-use (**D**) tips. *S*_*a*_*/nm* average surface roughness, *S*_*q*_*/nm* surface mean square roughness.

**Figure 6:**
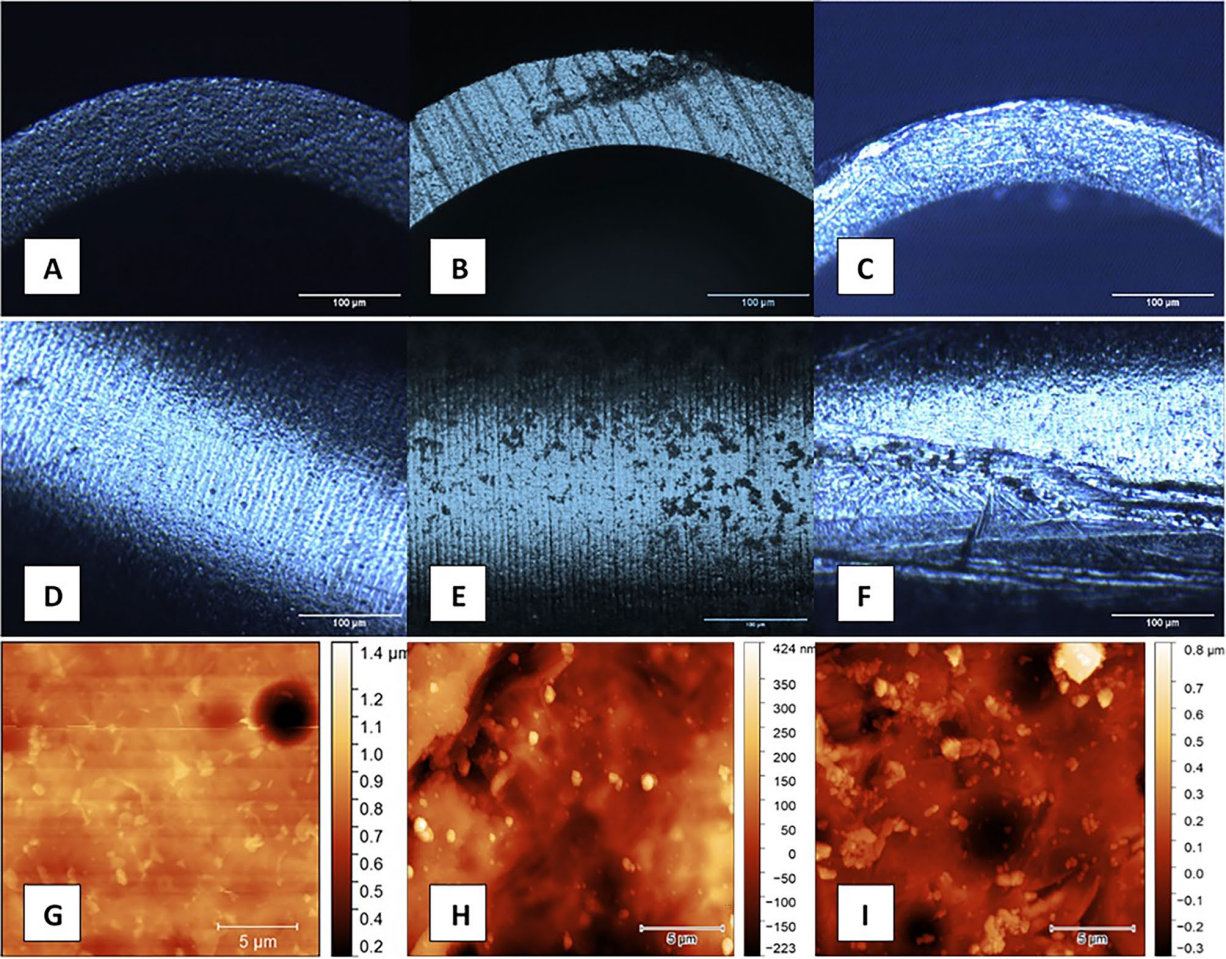
(**A**,**B**,**C**): Microscopic changes after fifty operations on the edge of a single-use (**B**) and a multiple-use tip (**C**). (**D**,**E**,**F**): Microscopic changes after fifty operations on the mantle of a single-use (**E**) and a multiple-use tip (**F**). (**G**,**H**,**I**): Atomic-force microscopic changes after fifty operations on the edge of a single-use (**H**) and a multiple-use tip (I). A,D,G: Unused tip.

A gradual decrease in area % and an increase in profile length % were observed for both single-use and multiple-use tips, which is also confirmed by the change in roughness indicators (Figs. 5, 6).

The analysis of the data revealed that the roughness of the samples increased significantly after 5 operations, which shows a gradual decrease with further use, and then for both tip types, a jump is visible after the twentieth operation, which is followed by another gradual decrease.

## Discussion

There are many publications on the use of atomic force microscopy in ophthalmic research. Research groups have investigated the aquaporin level in different areas of the lens during cataract formation, the structure of the lens and its changes, the trabecular network deviations in glaucoma, and the morphological and functional mapping of many constituent elements of the retina^6–11^. Berry et al. investigated mucin adhesions on the ocular surface, thus contributing to the understanding of the etiology of dry eye syndrome^7^. Last et al. investigated the mechanical properties of the human corneal basement membrane^8^. Ziebarth et al. examined the elasticity of the lens on monkey eyes^9^. Guo et al. examined the melanosomes in the retinal pigment epithelium using an atomic force microscope^10^. The atomic force microscopic examination of phacoemulsification tips was carried out for the first time in this study.

Phacoemulsification tips have been examined from several aspects before. Tsaousis et al. examined and compared unused, single- and multiple-use tips of several manufacturers using energy-dispersive X-ray spectroscopy, X-ray photoelectron spectroscopy and 3-dimensional white-light interferometry. In their results, they reported that the factory tips of different manufacturers differ in their oxygen and titanium content, however, they did not find any significant difference in the roughness of single- and multiple-use unused tips^1^. They then tested the effect of sterilization on unused tips. In their study, using a scanning electron microscope and an energy-dispersive X-ray spectroscope, they examined single- and multiple-use and reusable tips through several sterilization cycles. First after using an autoclave and detergents without rinsing between cycles, then the same processes with sterile water rinsing between cycles. Finally, tips sterilized without the use of detergents were examined. It should be highlighted from their results that, in addition to the use of detergents, with rinsing between cycles, far fewer deposits were visible on the surface of the tips. However, without the use of detergents, only by rinsing, the number of deposits on sterilized devices is almost negligible^2^. In the third study of the group, 8 different tips from 3 manufacturers were examined with scanning electron microscopy and white light interferometry. In their study, phacoemulsification was performed for 2 min with maximum (100%) phaco energy on porcine lenses of different degrees of maturity in an ex vivo animal experiment, and then this was repeated in 5 cycles. An increase in roughness was observed on different tips, but this change did not prove to be significant^3^. In our case, the tests were carried out in vivo, under normal surgical conditions, which already caused significant changes on the surface of the tips. As a result of the applied sterilization, we did not see any significant surface abrasion either.

Demircan et al. investigated, during torsional and transversal phacoemulsification, the effect of multiple-use tips on various surgical parameters and postoperative morphological differences. In their study, unused tips were compared with tips that were used ten or twenty times. During transversal phacoemulsification, neither the effective phaco time nor the total US time was found to be significantly different in any degree of cataract maturity. However, during longitudinal phacoemulsification, the reduction of total US time and cumulative dissipated energy (CDE) was clearly demonstrated during the operation of grade 3 and 4 cataracts when new tips were used. However, there was no difference between the pre- and postoperative central corneal thickness and endothelial cell density^5^. In our study, in addition to the various surgical parameters, tests were also performed to determine the degree of surface abrasion and microscopic damage of the phacoemulsification tips, and in addition to cataracts of grade 3 and 4, cataracts with a lower density were also operated.

Cecchini et al. investigated in vivo the abrasion of phaco tips and the deposits that accumulate after one or more operations. With X-ray photoemission spectroscopy, energy-dispersive X-ray spectroscopy, scanning electron microscopy and contact polymetry, it was shown that, as a result of surgery, a significant increase in roughness was visible on multiple used tips, and the initial titanium alloy coating of the tip changed to a great extent, and numerous deposits accumulated on the surface of the tips^4^.

The importance of our study lies in the fact that it is the largest study conducted in vivo under real surgical conditions.

In order to carry out our examinations, in addition to reflection light microscopy, we looked for an examination procedure that can image the samples at an even higher resolution compared to the previous studies recorded in the literature. For this reason, we chose atomic force microscopy, because it works at a resolution of 0.01 nm. As a result, deviations that could not be detected by previous test procedures became testable and visible. Another big advantage is that the sample to be tested does not require special preparation, so there are no artifacts that may form during the test and mask the result. Examining the samples at a higher resolution (< 1.0 nm) made it possible to reveal the damage of a fraction of a nanometre on the surface of the phacoemulsification tips, which had not been examined in this range until now. This may be the explanation for the fact that, in contrast to previous studies, a greater increase in roughness and surface microscopic damage was observed on the tips used with the chop technique^1–5^.

Contrary to studies and reports in the literature, our results also show that with the divide and conquer technique, less average US energy was needed during the surgeries than in the case of the chop technique^12–15^. This can be explained by that the majority of the studies examined parameters during cataract surgery with a higher degree of maturity, while in our case, patient selection was done randomly from all maturity levels, therefore, on average, grade 2 and 3 cataract operations were performed. Based on previous claims, the chop technique is the choice for 3rd and 4th grades.

In the present study, during the evaluation of the optical recordings, it was revealed that the roughness on the surface of the devices, i.e. the number of microdamages, increases with the number of uses. In all cases, the change is greater until the twentieth operation, and from then abrasion of the tips is smaller. This can result from scratches and damage on the surface of the needles, as well as their abrasion and possible refilling. Materials filling surface gaps and injuries (such as proteins or carbon-containing materials) can mask the extent of the injury, so their enzymatic or mechanical removal may also be considered future examinations to determine whether these deposits affect the safety of use or the development of complications.

Based on all the above, it can be concluded that the more times a tip is used, the more its surface is damaged, but perhaps only up to a certain number of operations. During the examination of the different tips, we also saw that the type of tips has no influence on the degree of abrasion, so there is no significant difference between single- and multiple-use tips in terms of abrasion. Thus, the multiple use of single-use tips should be considered, from, among other things, cost-effectiveness point of view. Currently, the manufacturer recommends performing a maximum of 10 surgeries with a multiple-use tip. Based on our results, however, the use of the tips 20 times should be considered, since this number of operations seems to be a certain limit in terms of abrasion of the tips.

From our results, it is also clear that in the case of less hard lens nuclei, the needle surface is less damaged with the divide and conquer technique, and less average US energy is required during surgery. Due to these results, the use of the divide and conquer surgical technique seems to be more reliable for cataract surgeries of this level of maturity, in terms of tip abrasion.

The results may help to understand the development of wear processes in different surgical techniques, which may influence subsequent manufacturing technology modifications. How the quality and quantity of these wear processes may affect intraoperative surgical complications could be the basis for further research.

The clinical significance of the results can also be significant because by increasing the use of tips, less surgical waste is generated, which unfortunately is an increasing problem in today's world. In this way, ophthalmologists can also contribute, to some extent, to the improvement of global waste management and to the reduction of increasing environmental pollution.

## Data Availability

The data that support the findings of this study are not openly available due to reasons of sensitivity and are available from the corresponding author upon reasonable request. Data are available at data storage of University of Debrecen, Clinical Center Kenezy Gyula Campus, Department of Ophthalmology.
